# Immunity, suicide or both? Ecological determinants for the combined evolution of anti-pathogen defense systems

**DOI:** 10.1186/s12862-015-0324-2

**Published:** 2015-03-13

**Authors:** Jaime Iranzo, Alexander E Lobkovsky, Yuri I Wolf, Eugene V Koonin

**Affiliations:** National Center for Biotechnology Information, National Library of Medicine, National Institutes of Health, Bethesda, MD 20894 USA

## Abstract

**Background:**

Parasite-host arms race is one of the key factors in the evolution of life. Most cellular life forms, in particular prokaryotes, possess diverse forms of defense against pathogens including innate immunity, adaptive immunity and programmed cell death (altruistic suicide). Coevolution of these different but interacting defense strategies yields complex evolutionary regimes.

**Results:**

We develop and extensively analyze a computational model of coevolution of different defense strategies to show that suicide as a defense mechanism can evolve only in structured populations and when the attainable degree of immunity against pathogens is limited. The general principle of defense evolution seems to be that hosts do not evolve two costly defense mechanisms when one is sufficient. Thus, the evolutionary interplay of innate immunity, adaptive immunity and suicide, leads to an equilibrium state where the combination of all three defense strategies is limited to a distinct, small region of the parameter space. The three strategies can stably coexist only if none of them are highly effective. Coupled adaptive immunity-suicide systems, the existence of which is implied by the colocalization of genes for the two types of defense in prokaryotic genomes, can evolve either when immunity-associated suicide is more efficacious than other suicide systems or when adaptive immunity functionally depends on the associated suicide system.

**Conclusions:**

Computational modeling reveals a broad range of outcomes of coevolution of anti-pathogen defense strategies depending on the relative efficacy of different mechanisms and population structure. Some of the predictions of the model appear compatible with recent experimental evolution results and call for additional experiments.

## Background

The arms race between pathogens and hosts is a major force in evolution that is likely to have played key roles in the origins of multicellularity and sexual reproduction, among other biological phenomena [1-5]. In this race, hosts (both prokaryotic and eukaryotic) have evolved a broad repertoire of defense strategies, sometimes at the expense of allocating substantial resources and genomic space for defense functions [6-11]. Although a vast diversity of defense mechanisms exists across different life forms, the defense strategies can be generally classified into three groups [12]: (i) innate immunity systems that are based on recognition of conserved features of pathogens; (ii) adaptive (acquired) immunity systems that provide enhanced efficiency through pathogen-specific (Lamarckian-type) adaptive evolution [9,13,14]; and (iii) suicidal systems that cause programmed cell death (PCD) preventing further spread of the infection [15-17]. In principle, the PCD strategy can be considered a form of innate immunity given the apparent lack of specificity to particular pathogens in this form of defense [18,19]. However, given the substantial differences between the outcomes of immune response and programmed cell death for host, we adhere to the threefold classification of defense strategies for the purpose of the present analysis.

In prokaryotes, innate immunity involves, among other forms, receptor masking, restriction-modification (R-M), Argonaute-based RNAi-like machinery and DNA phosphorothioation systems [7,20-22]. Adaptive immunity that until recently had been considered a distinctive innovation in animals, is represented in prokaryotes by the CRISPR-Cas systems that are found in nearly all archaea and many bacteria [23-25]. Suicide (PCD) mechanisms comprise toxin-antitoxin (TA) and abortive infection (Abi) systems [26-28]. Although most of these defense mechanisms are widely distributed across the prokaryotic world, the prevalence of each class greatly varies among taxa [7]. Moreover, genes encoding different defense systems are often clustered in genomic islands, suggesting that some of them might function in association [7,29]. In particular, most CRISPR-Cas loci as well as many R-M loci contain genes encoding predicted toxins, leading to the hypothesis of a functional coupling between immunity and suicide or dormancy [7,12,30,31]. The diversity, intricacy and patchy distribution of anti-pathogen resistance mechanisms imply a highly complex evolutionary history so that unraveling the causes for the presence or absence of particular defense systems in individual taxa becomes a difficult task.

The fate of defense systems in a host population depends on multiple factors and trade-offs that contribute to the cost-benefit balance [32-35]. On the one hand, the benefits of having such systems depend on their efficacy and the frequency of infections. Efficacy itself is the outcome of the evolutionary arms race with pathogens, and in the case of CRISPR-Cas, mathematical modeling suggests that the efficacy of protection negatively correlates with virus diversity and host and virus effective population sizes [36,37]. On the other hand, fitness costs can vary for different systems and depend on physical and ecological variables, such as temperature, the identity of the pathogens targeted by the resistance mechanisms and the relevance of horizontal transfer for the acquisition of beneficial genes [38-43]. Population structure also likely plays a role in the evolution of defense systems, not only through the repercussions for the ecological pathogen-host dynamics [44], but also via direct and indirect effects on the efficacies of different forms of defense [45]. Such effects are well illustrated by experiments with a genetically engineered suicidal system in *Escherichia coli* which proved to be a successful phage defense mechanism in soft agar culture but not in liquid medium [46]. Fully compatible conclusions on the essential, general role of population structure for the evolution and maintenance of programmed cell death have been reached by mathematical modeling [4]. Finally, pre-existing resistance could affect the evolution of additional defense systems [47]. Specifically, the question ‘Why evolving two costly systems for a single task?’ applies to the evolution of suicide in the presence of immunity, especially because suicide can be interpreted as a defense system with an infinite individual fitness cost.

Here we develop and analyze a computational model aimed at understanding which types of defense mechanisms evolve under distinct ecological conditions. We examine a scenario where hosts already possess some degree of resistance to pathogens via innate immunity and can evolve additional, costly immunity and/or suicide mechanisms. By exploring a broad range of immune and suicide efficacies in combination with variable degrees of population structure (from a well-mixed population to a fully structured population with no migration), simulations reveal a spectrum of evolutionary behaviors characterized by the acquisition of one, two or all three classes of defense mechanisms. While population structure proves critical for the evolution of cell suicide, acquisition of immunity mostly depends on its efficacy. We also extended the model to investigate possible causes and consequences of coupling between adaptive immunity and suicide. Taken as a whole, these results provide context to the co-occurrence of defense mechanisms in nature.

## Methods

### The computational model of pathogen-host coevolution

We set out to model a population of hosts and pathogens embedded in an interaction network. Such a network determines not only the local dispersion of the pathogen but also the possible locations of a host’s offspring. In addition, long-range dispersion of hosts and pathogens to random locations on the network is allowed at certain mixing rates. As the long-range dispersion rates increase, the structured system gradually gives way to a well-mixed one. Two types of interaction networks were implemented: (i) a square grid resembling a 2-dimensional spatial structure, and (ii) a community network in which hosts are structured in a number of tightly connected clusters (cliques) of a given size. In the latter case, clusters other than self can only be accessed through long-range dispersion of host and pathogen. Only one host per site is allowed. For computational purposes, the number of pathogens per site is limited to 1000 (although in practice such a high number is not reached). In the community network, group sizes ranging from 2 to 100 totally connected sites per group were explored through separate simulations. The network size, which determines the carrying capacity for hosts in the simulations, is equal to 10^6^.

Ecological and evolutionary dynamics come into play through the following processes:

- (1) Host reproduction at a rate *g*, which succeeds if a randomly chosen neighbor site in the network is empty. In such a case, a copy of the parent host is placed in that site.
- (2) Host death at a rate *d*_*host*_
- (3) Infection at a rate *b* * *n*_*path*_ where *n*_*path*_ is the number of pathogens sharing the site with the host. Infection immediately leads to three possible outcomes:
- (3a) Host survives and no pathogen is produced (efficient immunity).
- (3b) Host dies and no pathogen is produced (efficient suicide).
- (3c) Host dies and *M* pathogens are produced (efficient infection).

The outcome of the infection depends on the immune efficacies (*pII* for innate immunity, *pAI* for adaptive immunity, *pCD* for suicide) and it is decided a sequential way: first, an innate immune response is tried. If it fails (which occurs with probability 1 − *pCD*), adaptive immunity comes into play. Suicide can be attempted before or after immunity; the differences between the two regimes are addressed when relevant. Because either immunity, suicide or pathogen release occurs right after the infection there is no reproduction or dispersion of infected hosts.

- (4) Pathogen degradation at a rate *d*_*path*_.
- (5) Pathogen diffusion to neighbor sites at a rate *D*.
- (6) Population mixing: the contents of two randomly chosen sites are swapped at rates *rmix*_*host*_ (for hosts) and *rmix*_*path*_ (for pathogens). A fully structured scenario is set by making both rates equal to zero in the square grid network and to 0.01 in the community network. Notice that the latter requires a (small) value greater than zero in order to avoid total isolation of groups, which would lead to a population dynamics entirely driven by small-size effects, with fast and permanent extinction of parasites and/or hosts in separate groups.

The model is implemented through a simplified version of the tau-leaping algorithm. First, a time step *dt* is set by taking the inverse of the largest event rate and multiplying it by 0.2; then *per individual/per site* probabilities for host reproduction, host death, infection, pathogen degradation and mixing events are calculated as *p*_*event*_ = 1 − *e*^− *rate* × *dt*^. Notice that, given our choice of *dt* and the parameter values, the probability of the same event affecting the same individual (host or pathogen) more than once per time step is negligible. In the case of diffusion the probability that a pathogen drifts a given distance is taken from a Poisson distribution with mean equal to *D* × *dt*. After calculating event probabilities, we proceed through every site in the network in a random order. At each site, host replication and death are tried first, sequentially followed by infection, pathogen degradation and pathogen diffusion. The number of pathogens that are degraded is chosen from a Poisson distribution with mean *p*_*degr*_ × *n*_*path*_, where *n*_*path*_ is the number of pathogens in the site after the outcome of a possible infection. The number of pathogens diffusing a given distance is chosen in a similar way and their final locations are independently decided. Once all sites have been updated, a number of mixing events for hosts and pathogens is drawn from a Poisson distribution with mean *p*_*mix*_ times the number of sites in the network. Each mixing event is performed by randomly choosing two sites and exchanging their (host or pathogen) contents. This completes a cycle of the algorithm, which continues by setting a new time step and iterating the previous actions.

The population is initialized with suicide propensity equal to zero. Each time a host replicates, mutations leading to small changes in the suicide propensity of the daughter cell can take place with probability *μ*. Mutations are implemented by randomly choosing a value from a uniform distribution in the interval (−*ε*, *ε*) and adding that to the suicide propensity *pCD*. Suicide propensity is kept bounded between 0 and 1. The probability of successful suicide is the product of the suicide propensity and a parameter *CD*_*max*_, which represents the maximum possible efficacy for suicide. In the simulations, parameters *μ* and *ε* take values 0.01 and 0.1, respectively; the outcome of the simulation was shown to be highly robust to changes in the values of these parameters.

With respect to immunity, two or more classes of hosts with constant values of *pII* and *pAI* compete for fixation. Adaptive immunity (AI) is envisioned as a trait that provides the host with enhanced immunity at an additional cost. Under the assumption that there are different timescales for immune adaptation (fast) and gain/loss of AI-associated genes (slow), it is possible to assign a time-averaged efficacy to the adaptive immunity system and therefore circumvent explicit modelling of its “Lamarckian” adaptive dynamics. The immune efficacies *pII* and *pAI* should be interpreted as effective parameters, i.e. averages along an ecologically meaningful timespan which is long enough to allow for a large number of host-pathogen encounters but short compared to the evolutionary timescales involved in the acquisition and loss of innate and adaptive immunity systems. Mutations leading to AI acquisition or loss are set to occur with probability *λ* = 0.001. The fitness cost of adaptive immunity, *c*, is modeled as a death rate increase. This fitness cost is paid even if adaptive immunity is not activated (notice that the case where immune activation entails an additional fitness cost is formally equivalent to the variant of the model explored in section “Programmed cell death dependent on immunity”).

Simulations run for 4000 host generations or until hosts or pathogens become extinct. As a preliminary step, an extensive exploration of the parameter space was carried out in order to determine which parameter combinations allow for long-term coexistence of pathogens and hosts. Among those, the following base parameter values were selected: *g* = 1.01, *d*_*host*_ = 0.01, *M* = 30, *b* = 1, *d*_*path*_ = 2, *c* = 0.2, *D* = 10. To test the robustness of the results, several other parameter combinations within the coexistence space were also tried, with no qualitative changes. Also, ncreasing *λ* to 0.01 (so that mutation rates for suicide and adaptive immunity are the same) or *ε* to 0.5 (so that the effect of single mutations on suicide is larger) produced neither qualitative nor significant quantitative differences in the stationary state. Multiple combinations for parameters *pII*, *pAI*, *pCD* and *CD*_*max*_ were explored; the dependency of the results on their values is discussed along the text and the actual values used to produce the figures are specified in the figure captions. All simulations were performed using a C++ implementation of the model and the results were processed and analyzed with MATLAB (version R2014a, © 2014 The MathWorks, Inc.).

## Results

### Evolution of suicide in the absence of adaptive immunity

**Figure 1:**
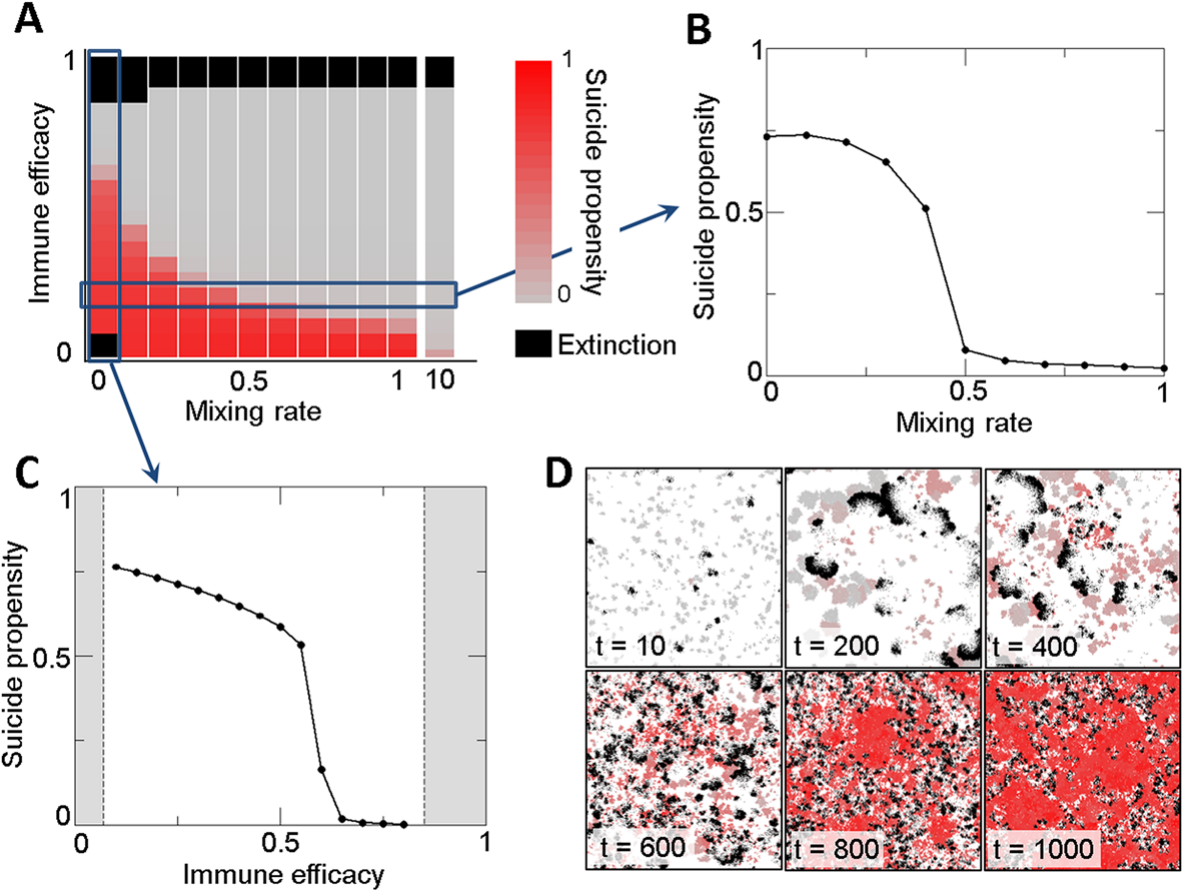
**Evolution of altruistic suicide in the absence of adaptive immunity. A**, **B**, **C**: Average suicide propensity of a population after the evolutionary process as a function on the efficacy of innate immunity and the population mixing rate. The color code in A indicates the mean suicide propensity. B shows results for varying immune efficacy in a fully structured population (mixing rate equal to 0). C shows the effect of weakening the population structure at a fixed immune efficacy equal to 0.2. **D**: Snapshots of the evolutionary process for an immune efficacy of 0.2 and no mixing. Grey to red colors indicate the suicide propensity of a host (as in the color bar in A), white denotes empty sites and black infected hosts. In this setting, the decision to commit suicide occurs before immunity. Maximum suicide efficacy *CD*_*max*_ = 1.

In order to simplify analysis of the model, evolution of suicide is considered first in the absence of adaptive immunity. To this end, all hosts are deprived of adaptive immunity (and its associated cost) from the beginning of the simulation and are not allowed to recover it. In these conditions, altruistic suicide evolves if (i) the efficacy of innate immunity is low or moderate, and (ii) the population is spatially structured (Figure 1). The first of these conditions is relaxed if suicide is only triggered once immunity has failed. In such a case, suicide becomes a non-costly trait (there is no cost for committing suicide if the host has no chance of survival). This arguably unrealistic behavior disappears if an explicit fitness cost is considered for suicide (for instance, an increased basal mortality associated with accidental induction of suicide in the absence of pathogen). Other parameters of the model, such as the infection rate (*b*) or the pathogen offspring number *M*, are relevant to determine the regions of the parameter space where hosts and pathogens can coexist but their relevance for the evolution of suicide is minor. Similar results were obtained with both the two-dimensional model and the community model. The case of different mixing rates for hosts and pathogens was also explored, with the result that evolution of suicide is much more sensitive to the long-range dispersion of hosts. As an example, for an immune efficacy of 0.2, suicide does not evolve if the host mixing rate is above 0.45 whereas the pathogen mixing rate can increase up to 9.0 without precluding suicide.

Figure 1D shows that evolution of altruistic suicide is concomitant with the formation of persistent islands of interacting individuals. The two-dimensional setting reveals that hosts self-organize in spatial islands that survive the longer the greater their suicide propensity. In the absence of suicide, waves of pathogen periodically clear out groups of hosts which cannot persist for a long time. However, as the suicide propensity evolves to higher values, host islands become more and more “impermeable” to the pathogen (i.e. suicide of the surface cells protects the inside of the island from the pathogen) which promotes persistence of such islands. In the two-dimensional setting, this effect is accompanied by a tendency of islands with high suicide propensity to grow and invade the available space.

Complementary to the spatially extended setting, the community model provides for exploration of the effect of host group size on the evolution of altruistic suicide. Under this model, hosts are placed in semi-isolated groups of limited size that locally behave as well-mixed populations, migration across groups taking place at a low rate. For small groups (up to at least 20 hosts per group), we did not find any relevant differences with respect to suicide evolution, the results being similar to those obtained in the two-dimensional grid. In contrast, simulations show that suicide does not evolve if groups contain about 100 hosts or more. For such large groups, the adverse effect of within-group well-mixing is stronger than the positive effect of the meta-population structure. As a result, large groups with high degree of phenotypic heterogeneity require internal structure to prevent exploitation by ‘selfish’ hosts and maintain altruistic suicide as an efficient defense mechanism.

**Figure 2:**
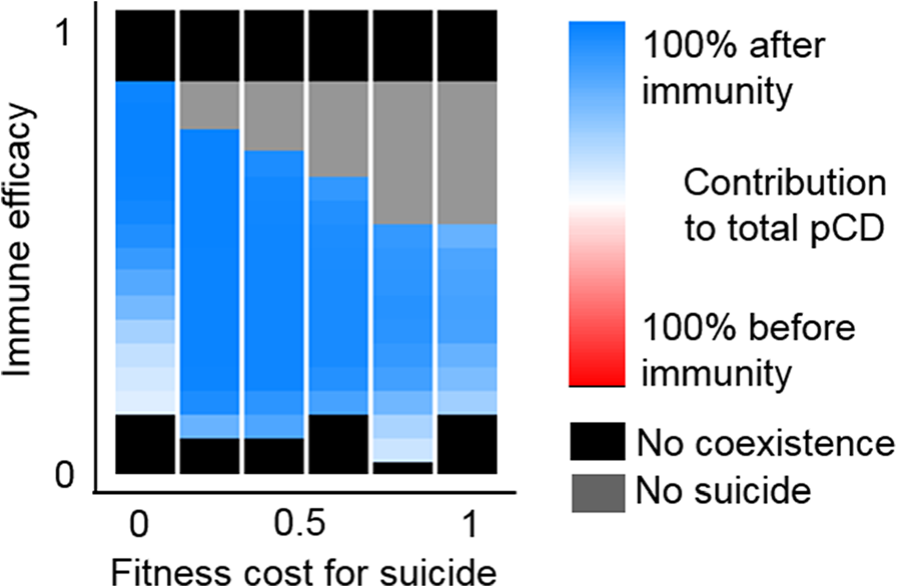
**Suicide after immunity failure is favored compared to suicide before immunity.** The color code, from red to blue, indicates the relative contribution of suicide before and after immunity to the total suicide propensity, provided that both strategies are equally accessible to the host. Horizontal and vertical axes correspond to different values of the immune efficacy and the cost of maintaining suicide, respectively. Cases of ‘no suicide’ correspond to final suicide propensities below 0.1. Fully structured population with maximum suicide efficacy *CD*_*max*_ = 1.

Finally, we explored a scenario where the choice between suicide and immunity is a selectable trait. Suicide before and suicide after immunity were treated as two alternative strategies that evolve independently and are equally accessible to the host. Under conditions that are favorable to the evolution of suicide, suicide after immunity always outcompetes suicide before immunity so that the final state of the population fully consists of hosts in which suicide is triggered only when immunity fails (Figure 2).

### Evolution of adaptive immunity in the absence of suicide

**Figure 3:**
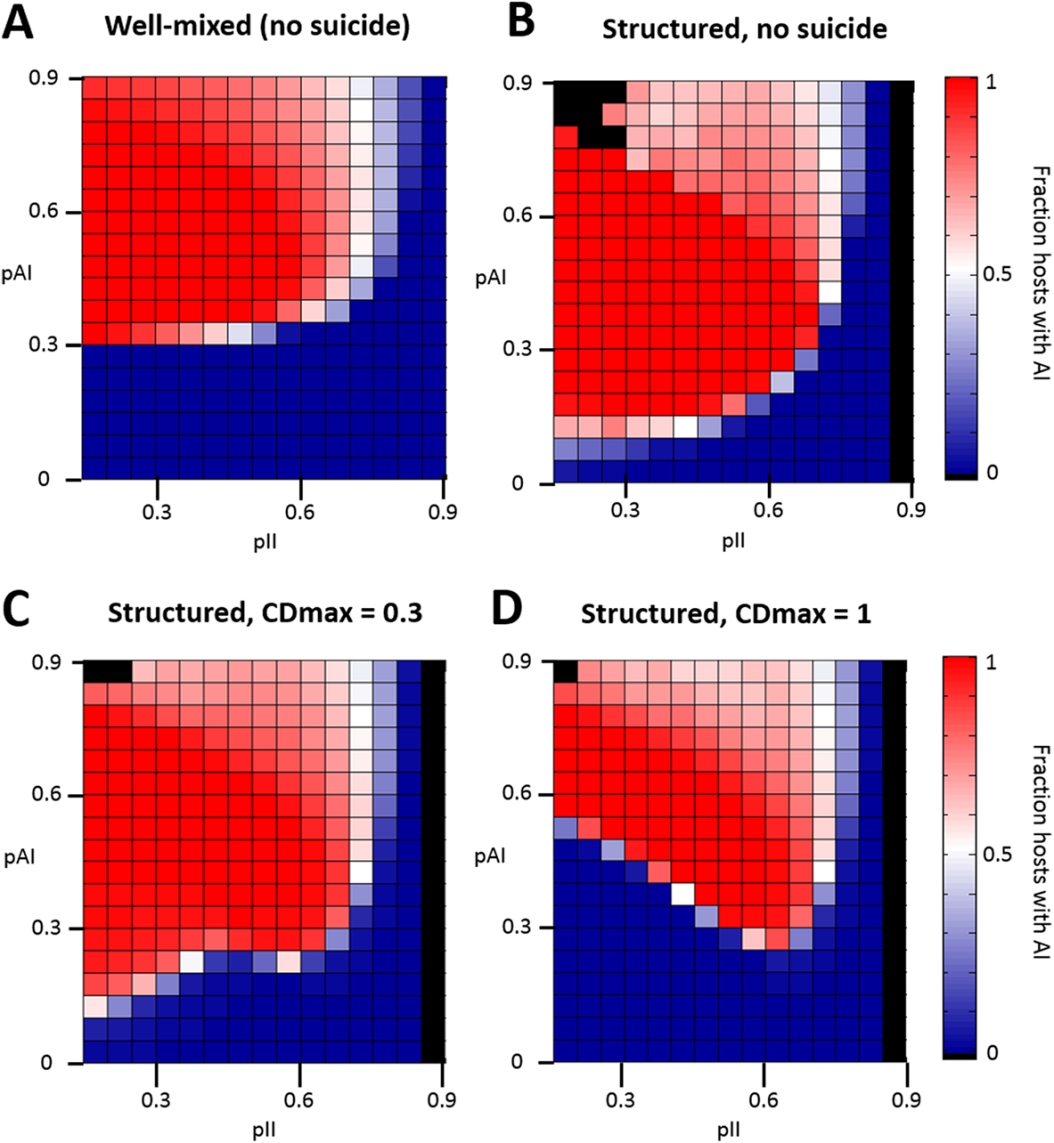
**Fixation of adaptive immunity only takes place for a certain range of immune efficacies.** The evolutionary process takes place at fixed values of innate and adaptive immune efficacies and the fraction of hosts with adaptive immunity is recorded in the stationary state. The. x-axis corresponds to the (externally fixed) efficacy of the innate immunity; the y-axis to the (externally fixed) efficacy of adaptive immunity; the color code indicates the fraction of hosts with adaptive immunity. Regions in red indicate fixation of adaptive immunity, while those in blue denote its loss. Coexistence of hosts with and without adaptive immunity takes place in regions with lighter colors. The evolutionary process takes place in **(A)** a well-mixed scenario, **(B)** a structured population without possibility of suicide, and **(C, D)** a structured population with the possibility of evolving suicide, with a maximum suicide efficacies of *CD*_*max*_ = 0.3 **(C)** or *CD*_*max*_ = 1 **(D)**.

We further explored evolution of adaptive immunity in hosts with no suicide. In this setting, suicide propensity is set to zero, and two classes of hosts, with and without adaptive immunity, compete for the limited spatial resources. Both classes of hosts have the same level of innate immunity and only differ by the presence or absence of adaptive immunity the efficacy of which is kept constant during the simulations. Figure 3 (A,B) shows the final composition of the population as a function of the immune efficacies of the competing hosts. Three regimes can be observed: i) fixation of adaptive immunity, ii) coexistence of hosts with and without adaptive immunity, and iii) loss of adaptive immunity. Fixation of adaptive immunity is only possible at moderate efficacies of both innate and adaptive immunity. As intuitively expected, poorly performing adaptive immunity is lost because its benefits do not compensate for the associated fitness cost. In the other extreme, less intuitively, adaptive immunity cannot be fixed if its efficacy is too high. In this case, the concentration of pathogens becomes so low that hosts lacking adaptive immunity can survive by taking advantage of their higher fitness compared to the hosts possessing adaptive immunity.

The effects of population structure and fitness cost on the evolution of adaptive immunity are merely quantitative. In particular, population structure favors adaptive immunity and reduces the minimum efficacy that is required for it to reach fixation (Figure 3A). The likely causes of this effect are discussed below. Not surprisingly, the associated fitness cost has a direct effect on the maintenance of adaptive immunity, with higher costs requiring higher efficacy (not shown). This effect is especially pronounced in a well-mixed population; in a structured population, however, adaptive immunity can be fixed even at higher costs.

### Combined evolution of suicide and adaptive immunity

In the well-mixed system, suicide does not evolve and the results are the same as those described in the previous section for the case without suicide. In structured populations, the interaction of suicide and immunity results in a complex behavior that yields different combinations of the three defense mechanisms.

**Figure 4:**
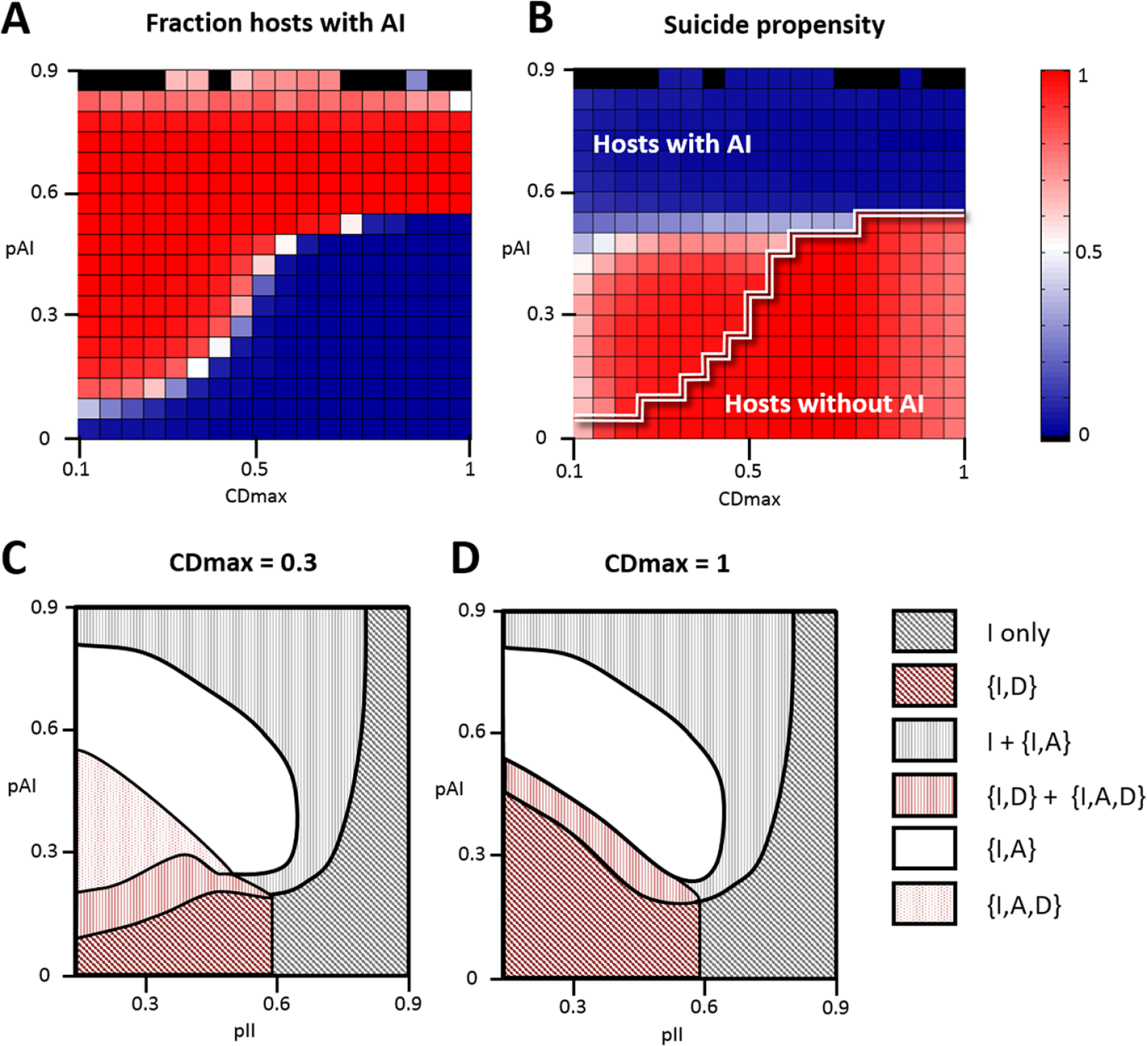
**Coevolution of adaptive immunity and suicide in a structured population depends on the immune and suicide efficacies. (A)**: Fraction of hosts with adaptive immunity in the stationary state of the evolutionary process, as a function of the AI efficacy (*pAI*, y-axis) and the maximum suicide efficacy attainable (*CD*_*max*_, x-axis). Red accounts for AI fixation, blue for AI loss. The II efficacy has been fixed to *pII* = 0.2. **(B)**: Suicide propensity evolved by majority hosts, in the same conditions as in A. Red accounts for high suicide propensities, blue for low or no suicide. The double white line separates two regions for which the majority hosts differ, in accordance to panel A. Notice that the actual suicide probability, *pCD*, is the product of the suicide propensity and the maximum suicide efficacy attainable, *CD*_*max*_. **(C, D)**: Phase diagrams for the combined evolution of suicide and adaptive immunity in a structured population. I: innate immunity; A: adaptive immunity; D: suicide; brackets indicate coevolution of traits within the same host; regions with a striped shading pattern present coexistence of hosts with different sets of defense mechanisms.

**Figure 5:**
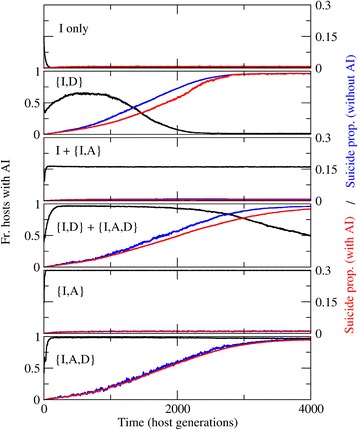
**Evolutionary dynamics of defense mechanisms, representatives of each of the six regions of the phase diagram.** Black: fraction of hosts with adaptive immunity; red: average suicide propensity in hosts with adaptive immunity; blue: average suicide propensity in hosts without adaptive immunity. Maximum suicide efficacy has been fixed to *CD*_*max*_ = 0.3, values of *pII* and *pAI* have been chosen from the phase diagram in Figure 4C to represent each possible evolutionary outcome. Population structured in a square grid with no mixing.

As schematically shown in Figure 4, different efficacies of the innate and adaptive immunities, as well as different values of the maximum suicide efficacy, give rise to distinct evolutionary outcomes which can be organized in a phase diagram with 6 regions (Figure 4 C, D). Suicide and adaptive immunity coevolve in a region ({IAD}) that typically corresponds to small and moderate efficacies of the innate immunity and moderate efficacy of the adaptive immunity, and only if there is also a limit on the efficacy of suicide. When the efficacy of adaptive immunity exceeds a certain threshold, suicide no longer evolves ({IA}). For even higher efficacies of adaptive immunity (I+{IA}), the evolutionary process gives rise to two divergent classes: hosts with adaptive immunity coexist with hosts that lack it, both exhibiting negligible suicide propensities. Notably, in the narrow coexistence region ({ID} + {IAD}), both classes of hosts, those with and without adaptive immunity, evolve high suicide propensities. In this region, the efficacy of adaptive immunity systems lies just below the threshold of fixation. In the long term, however, populations within this region might end up losing adaptive immunity (Figure 5). Finally, adaptive immunity is lost if either its efficacy is too low or the efficacy of innate immunity is too high. In the former case ({ID}), hosts evolve suicide as an additional defense mechanism; in the latter (I only), innate immunity is the only defense mechanism of the population. Temporal dynamics for representatives of each region are shown in Figure 5.

A comparison of the panels B, C, and D from Figure 3 makes it clear that evolution of suicide can preclude fixation of adaptive immunity. Specifically, as suicide evolves to higher efficacies (i.e. larger values of the parameter *CD*_*max*_), fixation of adaptive immunity becomes restricted to an increasingly narrow region of the parameter space. Figure 4A, B shows how increasing *CD*_*max*_ results in higher AI efficacies being required for AI fixation and a concomitant shrinkage of the region in which AI and suicide coevolve. A detailed analysis of such instances reveals a transient two-stage dynamics (Figure 5, {ID} and {ID} + {IAD}): first, adaptive immunity spreads in the population getting relatively close to fixation; subsequently, altruistic suicide evolves in a minority of hosts that lack adaptive immunity. Eventually, these individuals pervade the population and adaptive immunity is lost.

**Figure 6:**
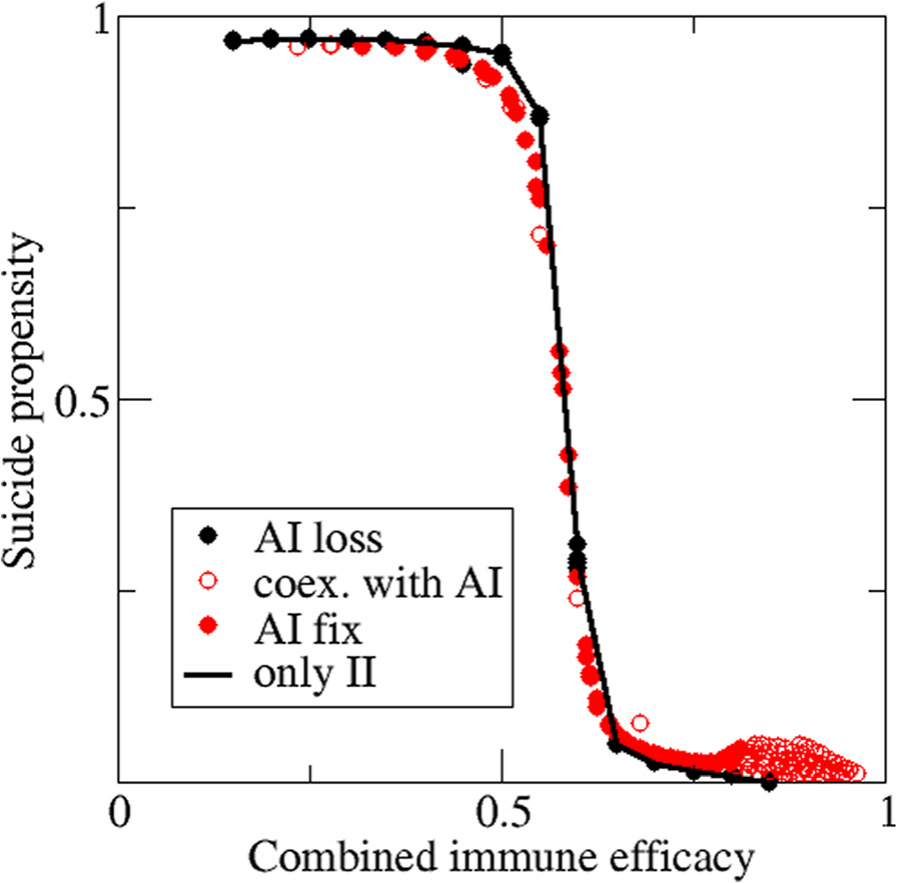
**Adaptive immunity does not affect the evolution of suicide.** The effective immune efficacy combines the efficacies of innate and adaptive immunities in a single parameter, so that results can be compared to the case with no adaptive immunity (solid line). Black circles correspond to instances that lead to the loss of adaptive immunity; solid red circles are used for instances with fixation of adaptive immunity; open red circles for hosts with adaptive immunity in instances without fixation. Data collapsed for structured populations with multiple values of *pII* and *pAI*; *CD*_*max*_ = 0.3.

We further tested whether adaptive immunity interferes with the evolution of suicide. To this end, combined immunity was defined as a measure of the combined efficacy of innate and adaptive immunity. Because both forms of immunity act sequentially and independently, the combined immune efficacy *pI*_*tot*_ equals to 1 − (1 − *pII*)(1 − *pAI*). Figure 6 shows the average suicide propensity versus the combined immunity for a set of simulations performed with different innate and adaptive immune efficacies. When adaptive immunity is lost, the results coincide with those obtained in the absence of adaptive immunity. Likewise, hosts with adaptive immunity (either fixed or in coexistence) show suicide propensities similar to those expected for equivalent immune efficacies in the absence of adaptive immunity. Therefore, it can be concluded that adaptive immunity does not specifically affect evolution of suicide as a defense mechanism.

The results presented in this section remain qualitatively the same regardless of whether suicide is activated before or after immunity, provided that a fitness cost for maintaining suicide is included (with zero cost suicide after immunity always evolves, whereas no changes are observed for suicide before immunity). The average suicide propensities might be slightly higher in the latter case but the regions of the phase diagrams (Figure 4C, D) remain similar.

### Programmed cell death dependent on immunity

**Figure 7:**
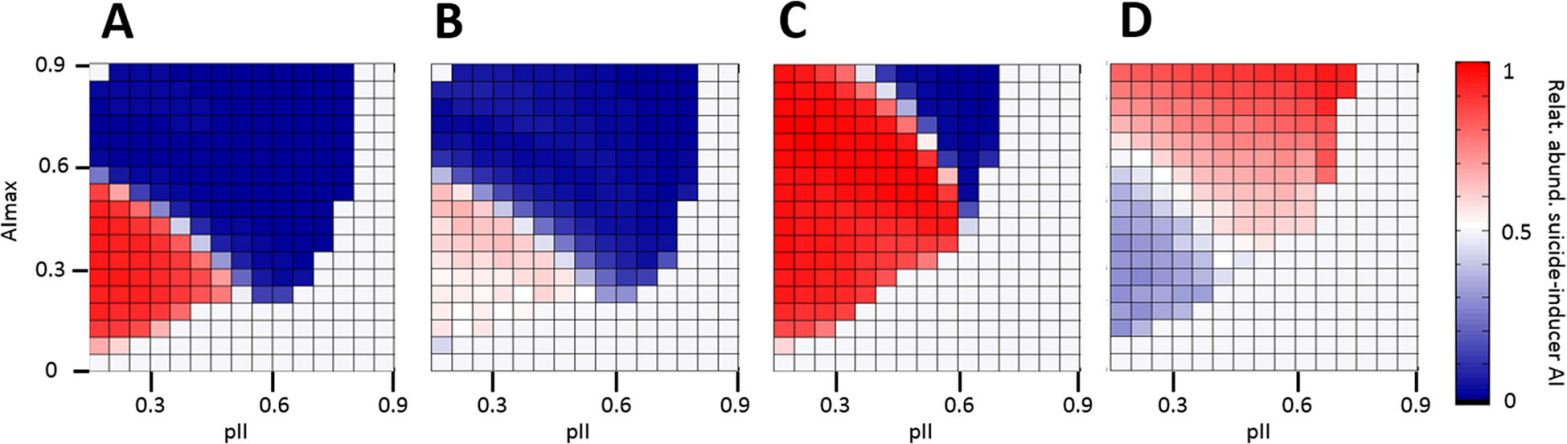
**Alternative models for the evolution of coupled suicide and immunity.** Two types of AI systems are considered: one whose activation can lead to cell death (suicide) with the probability *q* = 0.5, and another that only works as a standard immune mechanism (with no induction of additional suicide). Mutations between both types occur with a small probability. The figure shows the relative fraction of hosts with suicide-inducer AI in the stationary state (normalized by the fraction of hosts with any kind of AI, the value 0.5 has been assigned wherever hosts with AI represent less than 10% of the population). The evolutionary process was simulated in a structured population with different values of the innate and adaptive immune efficacies (*pII* and *pAI*), and a fixed value of the maximum suicide efficacy *CD*_*max*_ = 0.3. In **A**, the maximum suicide efficacy only affects AI-independent suicide; in **B**, the maximum suicide efficacy limits all kinds of suicide, included suicide induced by AI. Panels **C** and **D** correspond to a modified scenario where the efficacy of AI depends on the total degree of suicide according to a logistic formula; in C there is no limitation on AI-induced suicide, whereas in **D** it is limited by *CD*_*max*_. The red region in panel **D** (but not in other panels) is also characterized by the absence of AI-independent suicide.

Prokaryotic adaptive immunity systems, in particular CRISPR-Cas, encompass genes that are otherwise implicated in cell suicide such as toxin nucleases. In an attempt to shed light on the evolutionary factors behind such a tight co-occurrence of immunity and suicide, we examined a scenario where activation of adaptive immunity can lead to cell death and no pathogen production with probability *q*. This regime is equivalent to adding a new contribution to the suicide propensity of the form *q* * *pAI*. Two alternative formulations of the total suicide probability can be considered: on the one hand, if AI-dependent cell death is not affected by the maximum suicide efficacy, then *pCD*_*total*_ = *pCD* + *q* * *pAI* * (1 − *pCD*); on the other hand, if the maximum suicide efficacy *CD*_*max*_ also limits AI-dependent cell death, then *pCD*_*total*_ = *pCD* + *q* * *pAI* * (*CD*_*max*_ − *pCD*). In both cases, relatively large values of *q* are required to obtain significant contributions of adaptive immunity to cell death. We studied the possible outcomes of an evolutionary process where hosts can possess either an adaptive immunity system which does not induce suicide (*q* = 0, no AI-dependent suicide), or an adaptive immunity system which does induce suicide (*q* = 0.5, substantial AI-dependent suicide), or no adaptive immunity. The AI-independent suicide can evolve as described in the preceding sections; the probability of mutations leading to gain/loss of AI-dependent suicide was set at 0.001. The results shown in Figure 7 indicate that suicide-inducing immunity can be preferentially selected for in structured populations under conditions such that (i) coevolution of adaptive immunity and suicide is the expected evolutionary outcome and (ii) death induced by adaptive immunity is not limited by *CD*_*max*_. If AI-dependent death is limited by *CD*_*max*_, suicide-inducing adaptive immunity is transiently favored early in the evolutionary process but becomes a neutral trait compared to “simple” AI as soon as the probability of AI-independent suicide reaches values close to *CD*_*max*_. Finally, an adaptive immunity system that does not induce cell death is selected for in regions that are unfavorable to suicide evolution (this is also the case in well-mixed systems).

### Immunity dependent on cell death mechanisms

A distinct cause of the coupling between immunity and suicide could be that some of the functions performed by suicide genes are required for the immunity system to function as suggested, in particular, by the essentiality of Cas2, a homolog of toxin nucleases, for the CRISPR-Cas function [48], and presence of a number of other putative toxins in the CRISPR-cas loci [7,12,49]. Functional coupling between suicide and adaptive immunity can be introduced into the model by making the efficacy of adaptive immunity dependent on the suicide propensity. A simple logistic relation was chosen for this dependence *pAI*_*eff*_ = *AI*_*max*_ * *pCD*/(*pCD* + *kAI*). In this model *kAI* is a parameter that determines the shape of the dependence between suicide and adaptive immunity, and *AI*_*max*_ is the maximum possible value of *pAI*. For small suicide propensities, the effective *pAI* is equal to *AI*_*max*_ * *pCD*/*kAI*. If *pCD* ≫ *kAI*, the effective *pAI* tends to *AI*_*max*_. Given that *pCD* only takes values smaller or equal to unity, the maximum value that *pAI*_*eff*_ can take is *AI*_*max*_/(1 + *kAI*).

Functional coupling of adaptive immunity with suicide could result in the evolution of suicide under conditions where it would not normally evolve, e.g. in well-mixed populations or at high effective immunity levels. Moreover, relatively weak coupling (*kAI* = 0.5) is sufficient to observe these effects. To test whether functional coupling can explain the tight genomic co-occurrence of immunity and suicide genes, we use the logistic dependence of *pAI*_*eff*_ on *pCD*_*total*_ with the possibility that adaptive immunity contributes to *pCD*_*total*_, as in the previous section. Here we assume logistic dependence (functional coupling) and allow the adaptive immunity system to mutate between variants that do or do not induce additional death (*q* = 0.5 and *q* = 0, respectively). The same two cases as before, with *CD*_*max*_ limiting total suicide or AI-independent suicide only, were explored. Simulations for different values of *AI*_*max*_ and *pII* (Figure 7C, D) show that an adaptive immunity system that also induces death is strongly promoted if the suicide induced by AI is not limited by *CD*_*max*_. Conversely, if such a limit is in place, adaptive immunity systems that do not induce suicide tend to be selected for, provided that there is some degree of AI-independent suicide.

## Discussion

### General considerations on the modeling approach

In real biological communities, the efficacy of innate and adaptive immune systems is constrained by ecological factors such as population size, structure and diversity. The never-stopping arms race between hosts and pathogens sets a limit on the performance of defense strategies, which is determined by the ability of the host to keep the pace with pathogen escape mutations. In that regard, studies on host-parasite local adaptation suggest that parasites are usually ahead of hosts in this coevolutionary arms race [50,51]. Accordingly, evolution of immune systems is modeled here under the assumption that they have a constant efficacy that is extrinsically determined by a multiplicity of factors that go beyond the control of the host. In this simple model, the evolutionary process only determines the relative abundance of hosts with and without a given type of immune systems in the population. Nevertheless, even in this simple setting, the combination of different immune efficacies, population structure and long range dispersal leads to a complex interplay between different evolutionary outcomes which we aimed to explore here.

The notion of effective immune efficacies is valid under the assumption that there are two well differentiated timescales: (i) a relatively fast timescale for the processes of pathogen escape and host immune adaptation; (ii) a significantly slower timescale for the evolution (gain/loss) of defense mechanisms. Under this timescale separation, variations in immune efficacy due to the former processes can be considered transient perturbations around a time-averaged efficacy, which is the relevant value at longer evolutionary timescales. For the CRISPR-Cas system, this assumption implies that the characteristic timescales of viral proto-spacer mutation and host spacer incorporation are substantially faster than the timescale of CRISRPR-Cas loci gain/loss, an assumption that seems to be borne by the available observations [24,52]. We note that, because of timescale separation, the long term evolutionary dynamics of adaptive immunity can be studied by modeling adaptive immunity as a sort of stronger “innate immunity” with a higher cost. The same approximation would not be valid, though, to study short term, fine grained coevolution of hosts and parasites. A further limitation of our model concerns the immediate resolution of the infection event towards immunity, suicide or pathogen release. While this approach is especially suitable for modeling lytic infections, it does not account for the effects of persistent infections or milder parasites. In such cases, migration and/or reproduction of infected hosts should be taken into account, as well as possible differences in timing between different defense mechanisms. Indeed, theoretical studies in the former direction suggest that evolution of altruistic defense traits is hindered when infected hosts conserve some degree of reproductive ability [45].

The binary nature of the adaptive immunity trait in our model captures the qualitative difference between having and not having a functional CRISPR-Cas system. In contrast, pathogen-induced cell death (suicide) is modeled as a continuous trait such that, for a given host, the suicide propensity is the probability that a host attempts suicide when it becomes infected. Successful suicide leads to the death of the host and no pathogen release. Mutations affecting the suicide propensity can be interpreted as the result of variations in the copy number of toxin-antitoxins or other systems that contribute to cell death. Because a genome may contain a large number of loci involved in suicide [7], the relative effect of single mutations on the net suicide propensity are presumably small. By modeling programmed cell death as a quantitative trait, we capture this gradual nature of suicide evolution. Simulations explore the possibility that suicide gradually evolves starting from a population with zero suicide propensity. Provided that pathogens can also counter suicide mechanisms by inhibiting them or delaying their effect [6,53], it seems appropriate to include a maximum efficacy for suicide which limits the probability of successful suicide even if the suicide propensity is equal to one.

Throughout this analysis, we observed that population structure substantially affected the outcomes of pathogen-host interaction and evolution of defense mechanisms. In a structured population, hosts and pathogens are not randomly mixed in a homogeneous environment. Population structure can result from purely physical constraints (e.g. limited host and pathogen diffusion) or from the way individuals interact with each other (e.g. social networks). In order to account for both possibilities, the model was tested in two structured settings: a 2-dimensional grid with limited diffusion and a community network in which small groups of hosts closely interact with each other. The results were closely similar in both settings, confirming that population structure itself, regardless of its particular origin and implementation, is the cause of the observed behaviors.

### Evolution of different types of immunity and suicide

As shown in the preceding sections, suicide only evolves in structured populations for which long range dispersal remains under a threshold rate. Why is population structure required for the evolution of suicide? As with other altruistic traits, a host does not benefit from its own suicide. Instead, it provides a benefit to its neighbors by preventing the local spread of the infection. Therefore, suicide can evolve only when there is phenotypic relatedness among neighbors [54,55]. In a structured population, hosts with a high suicide propensity will be likely surrounded by other suicide-prone hosts and accordingly receive indirect benefit from the suicide of their neighbors [45]. Long-range dispersion, as well as population mixing, would bring together hosts with large and small suicide propensities. In a well-mixed population, the latter take advantage of the former so that suicide propensity becomes a disadvantage. From a game theory perspective, this dynamics defines a social dilemma where population structure allows altruistic hosts (those with high suicide propensity) to beat selfish ones (those with small suicide propensity) [56]. High rates of long-range pathogen dispersal can also preclude the evolution of suicide if local pathogen spread becomes irrelevant compared to the constant arrival of pathogens from the outside. Multiple experimental studies in bacteria have shown that population structure (low dispersal rate) is indeed essential for the evolution of cooperative traits [57-59] and in particular altruistic suicide [46,60]. It should be noted that, although not considered in our model, horizontal gene transfer could also promote evolution of altruistic suicide by increasing phenotypic relatedness between neighboring hosts [61].

The results of the present model of suicide evolution reveal an antagonism between the ‘interests’ of hosts and pathogens in structured populations whereby, while hosts tend to evolve suicide, prevention of host death is the preferred strategy for the pathogen. Indeed, it has been shown that experimental evolution of phages under conditions of local dispersion leads to ‘prudent’ (less virulent) variants of the virus, in contrast to the virulent forms evolved in a well-mixed environment [46]. Theoretical work also suggests that reduced virulence is the most likely evolutionary outcome under conditions of low pathogen dispersal, whereas intermediate dispersal rates can lead to high virulence [62]. It is therefore plausible that, in structured populations, the host-pathogen arms race has a major effect on the genes involved in cell death and its inhibition. Numerous viruses indeed have evolved mechanisms preventing host cell death, often by recruiting genes involved in host suicide and turning them into competitive inhibitors of cell death [63-65] or else via repair of the damage involved in cell death [66].

Adaptive immunity provides a direct benefit to the host organism that possesses the immune mechanism and thus does not require population structure to evolve. There are, however, instances when population structure favors fixation of adaptive immunity through a mechanism analogous to the one observed for altruistic suicide. Due to limited pathogen dispersion, hosts can benefit from the local decrease in pathogen abundance produced by their neighbors’ adaptive immunity. In a structured population, neighboring hosts are phenotypically correlated, reinforcing the advantage of adaptive immunity and contributing to its fixation. This effect could allow fixation of adaptive immunity even under conditions where the cost-benefit balance is not in its favor. On the other hand, if the immune efficacy is too high, hosts with and without adaptive immunity tend to coexist as the former generate environments with low pathogen pressure. This result resembles those obtained by previous theoretical studies on well-mixed populations, which predict lack of fixation for highly effective immunity systems and possible polymorphisms on the degree of immunity evolved by hosts [67,68].

Joint modeling of suicide and adaptive immunity gives rise to a distribution of evolutionary outcomes (a phase space) that cannot be simply explained through separate analysis of each of these defense mechanisms. For instance, high suicide efficacies prevent fixation of adaptive immunity not only in those regions where the cost-benefit balance for adaptive immunity is slightly unfavorable (even if fixation would occur in the absence of suicide by virtue of population structure), but also in some regions where this balance is favorable to adaptive immunity. There seems to be a rule of thumb whereby hosts do not evolve an additional, costly defense mechanism when one such mechanism is sufficient for effective defense. The most obvious manifestation of this simple principle is that, when innate immunity is highly effective, no other defense mechanisms evolve. Similarly, hosts with large suicide efficacy overtake those with adaptive immunity provided that the suicide propensity is greater than a threshold that grows with the efficacy of adaptive immunity. Notwithstanding this general trend, innate and adaptive immunity systems appear to coexist in other regions of the phase space. Such a coexistence is compatible with the recent explicit experimental demonstration of the synergy between R-M and CRISPR-Cas in bacterial resistance to bacteriophages [69]. Finally, there is a region of moderate immune and suicide efficacies where hosts take advantage of a combination of all three defense mechanisms: innate immunity, adaptive immunity, and suicide.

In an attempt to explain the tight connection between the CRISPR-Cas systems and (predicted) toxin genes [7,12,49], we explored the possible evolutionary advantage of an AI system which also induces suicide with a given probability. Such a coupled system can be advantageous if (i) suicide itself is advantageous in those particular evolutionary conditions and (ii) the efficacy of AI-associated suicide exceeds that of other suicide mechanisms. The same two conditions for the evolution of a coupled AI-suicide system were identified in a modified model that considers a small suicide propensity as a pre-requisite for the evolution of effective adaptive immunity (i.e. when components of the suicide machinery also contribute to adaptive immunity).

Host suicide can potentially evolve either as an immediate response to infection or as a means of last resort after immunity mechanisms fail to stop pathogen reproduction [12]. Intuitively, the second strategy would appear advantageous, and indeed in our model, when the two strategies evolve independently and then are allowed to compete, “suicide after immunity failure wins”. However, under a non-negligible cost of suicide, the probabilities of it evolving before and after immunity is tried are comparable. It is worth mentioning that, at cost zero, the conditions for the evolution of suicide after immunity become greatly relaxed. Thus, the cost of suicide appears to be a key determinant of the evolutionary regime. Recent experiments with suicide-prone *E. coli* have shown that at least when defense against highly virulent phages is involved, the cost of suicide could be quite low because the infected bacteria are moribund in any case and have virtually no chance to propagate [60]. Conceivably, these experiments reflect the regime with failing immunity and subsequent low-cost suicide.

### Implications for coevolution of prokaryotic defense mechanisms

Together with innate immunity systems, cell suicide (programmed cell death) and adaptive immunity are widespread defense mechanisms in prokaryotes although adaptive immunity is far from being universal. Two interpretations, not mutually exclusive, appear plausible:

- The efficacies of innate and adaptive immunity systems in prokaryotes lie in the appropriate region that allows for combined evolution of suicide and adaptive immunity. This view is compatible with the fact that adaptive immunity is not universal in prokaryotes and its presence or absence seems to be related to ecological variables (e.g. adaptive immunity is much more common in hyperthermophiles compared to mesophiles). Such a patchy distribution suggests that the mean efficacy of adaptive immunity is close to the boundary of the fixation region, with variation among ecosystems determining whether a particular host population maintains adaptive immunity or loses it.
- Different defense mechanisms are geared to fight different threats. Immunity systems might be highly effective against certain threats but less proficient against others. Suicide can serve as a defense mechanism that acts on those threats against which the immunity systems are less effective. This scenario accounts for the pre-requisite of the evolution of suicide revealed by the analysis of the models, namely that immune efficacy has to be low or moderate for suicide to evolve and vice versa.

Under both scenarios, population structure is required for the evolution of suicide. This pre-requisite had been previously proposed on the basis of experimental studies with a genetically engineered, suicidal strain of *E. coli* grown on agar plates [46] and is theoretically extended here to a broader spectrum of population structures, suicide efficacies and co-occurring immune mechanisms.

The presence of programmed cell death systems in the great majority of prokaryotes strongly suggests that structured populations are the rule in microbial ecosystems [4]. Indeed, some kind of social interaction, such as biofilm formation, is found on almost every bacterial group, and more advanced forms of sociality, such as quorum sensing, are often present in Proteobacteria and Firmicutes [70-72]. However, the link between PCD and sociality is complicated by the fact that toxin-antitoxin and Abi are addictive selfish elements, so that their abundance in bacteria and archaea is likely to be in part dictated by this property rather than by any utility to the host [26,27].

With respect to the presence of genes encoding (predicted) toxins in most if not all CRISPR-Cas loci, at least two, not necessarily mutually exclusive interpretations seem possible:

- The CRISPR-associated toxin genes contribute to the suicide propensity of the cell, in such a way that cells with CRISPR-Cas-toxin systems are more effective in orchestrating suicidal responses than those with “regular” suicide systems only.
- Suicide and adaptive immunity are functionally coupled, so that it is impossible to maintain an adaptive immunity system without previously developing other functionalities that involve suicide propensity. Although, under our model, coupled AI-suicide systems are not a necessary evolutionary outcome arising from this requirement alone, this could be the case if “regular”, non-AI-associated suicide systems were unable to provide the cells with the functionalities required to maintain a working adaptive immunity system. This possibility appears realistic given that evidence that Cas2 protein, a homolog of toxin nucleases, is essential for the adaptation stage of the CRISPR response although the nuclease activity of this protein apparently is not required. Further investigation of the molecular mechanisms that are responsible for the adaptation step of the CRISPR-Cas adaptive immunity is expected to clarify the nature of coupling between adaptive immunity and PCD.

## Conclusions

Evolution of suicide as a defense mechanism is only possible if (i) the host population is structured and (ii) the degree of immunity that the hosts can achieve by other means is limited. The widespread nature of suicide systems in prokaryotic genomes calls for a reconsideration of the relative importance of population structure for genome evolution.

Perhaps somewhat paradoxically, fixation of a costly adaptive immunity system is precluded not only when the innate immunity and suicide systems are highly effective but also when adaptive immunity itself performs at a high level. In such a case, hosts with and without adaptive immunity coexist in an environment where the pathogen is scarce (herd immunity effect).

Under the present model, the evolutionary interplay of suicide, innate immunity and adaptive immunity leads to an equilibrium state where the combination of all three defense mechanisms is limited to a distinct region of the parameter space. Such a region is smaller than it could be expected from studying suicide and adaptive immunity independently. This finding is consistent with the general principle that hosts do not usually evolve two costly defense mechanisms when one is sufficient.

There is, however, a range in the efficacies of suicide, innate and adaptive immunity systems that leads to combined evolution of suicide and adaptive immunity. This outcome is possible only when none of these defense systems is highly effective and requires some degree of structure in the host population. Therefore, it can be predicted that certain ecological factors such as pathogen diversity and effective population sizes may indirectly promote the evolution of alternative defense systems through limiting the effectiveness of prevailing ones.

Finally, coupled adaptive immunity-suicide systems can arise under two distinct scenarios: first, if the AI-associated suicide shows a better performance than other suicide systems; second, if the adaptive immunity component functionally depends on its associated suicide component and such functionality cannot be provided by independent suicide systems. Further research on the molecular biology of CRISPR-Cas systems and their associated toxin genes will be necessary in order to assess the feasibility and contribution of each of these scenarios.

The results of the present analysis most directly apply to the mechanisms of anti-pathogen defense in bacteria and archaea. However, the conclusions on the coevolution of different defense strategies suggest that the combination of immunity and/or suicide systems of multiple kinds is a general feature of evolving organisms. The patterns of co-occurrence of these mechanisms depend on ecological factors such as host and pathogen diversities and habitat structure.

## Abbreviations

Abi: Abortive infection
AI: Adaptive immunity
II: Innate immunity
PCD: Programmed cell death
R-M: Restriction-modification
TA: Toxin-antitoxin

## References

[1] Forterre P, Prangishvili D (2009). The great billion-year war between ribosome- and capsid-encoding organisms (cells and viruses) as the major source of evolutionary novelties. Ann N Y Acad Sci.

[2] Forterre P, Prangishvili D (2013). The major role of viruses in cellular evolution: facts and hypotheses. Curr Opin Virol.

[3] Koonin EV, Wolf YI (2012). Evolution of microbes and viruses: a paradigm shift in evolutionary biology?. Front Cell Infect Microbiol.

[4] Iranzo J, Lobkovsky AE, Wolf YI, Koonin EV (2014). Virus-host arms race at the joint origin of multicellularity and programmed cell death. Cell Cycle.

[5] Stern A, Sorek R (2011). The phage-host arms race: shaping the evolution of microbes. Bioessays.

[6] Labrie SJ, Samson JE, Moineau S (2010). Bacteriophage resistance mechanisms. Nat Rev Microbiol.

[7] Makarova KS, Wolf YI, Koonin EV (2013). Comparative genomics of defense systems in archaea and bacteria. Nucleic Acids Res.

[8] Rechavi O (2013). Guest list or black list: heritable small RNAs as immunogenic memories. Trends Cell Biol.

[9] Rimer J, Cohen IR, Friedman N (2014). Do all creatures possess an acquired immune system of some sort?. Bioessays.

[10] Boehm T (2011). Design principles of adaptive immune systems. Nat Rev Immunol.

[11] Boehm T (2012). Evolution of vertebrate immunity. Curr Biol.

[12] Makarova KS, Anantharaman V, Aravind L, Koonin EV (2012). Live virus-free or die: coupling of antivirus immunity and programmed suicide or dormancy in prokaryotes. Biol Direct.

[13] Bergstrom CT, Antia R (2006). How do adaptive immune systems control pathogens while avoiding autoimmunity?. Trends Ecol Evol.

[14] Koonin EV, Wolf YI (2009). Is evolution Darwinian or/and Lamarckian?. Biol Direct.

[15] Barber GN (2001). Host defense, viruses and apoptosis. Cell Death Differ.

[16] Koonin EV, Aravind L (2002). Origin and evolution of eukaryotic apoptosis: the bacterial connection. Cell Death Differ.

[17] Ameisen JC (2002). On the origin, evolution, and nature of programmed cell death: a timeline of four billion years. Cell Death Differ.

[18] Labbe K, Saleh M (2008). Cell death in the host response to infection. Cell Death Differ.

[19] Upton JW, Chan FK (2014). Staying alive: cell death in antiviral immunity. Mol Cell.

[20] Vasu K, Nagaraja V (2013). Diverse functions of restriction-modification systems in addition to cellular defense. Microbiol Mol Biol Rev.

[21] Swarts DC, Makarova K, Wang Y, Nakanishi K, Ketting RF, Koonin EV (2014). The evolutionary journey of Argonaute proteins. Nat Struct Mol Biol.

[22] Chen P, Jeannotte R, Weimer BC (2014). Exploring bacterial epigenomics in the next-generation sequencing era: a new approach for an emerging frontier. Trends Microbiol.

[23] Makarova KS, Haft DH, Barrangou R, Brouns SJ, Charpentier E, Horvath P (2011). Evolution and classification of the CRISPR-Cas systems. Nat Rev Microbiol.

[24] Barrangou R (2013). CRISPR-Cas systems and RNA-guided interference. Wiley Interdiscip Rev RNA.

[25] Barrangou R, Marraffini LA (2014). CRISPR-Cas systems: Prokaryotes upgrade to adaptive immunity. Mol Cell.

[26] Gerdes K, Christensen SK, Lobner-Olesen A (2005). Prokaryotic toxin-antitoxin stress response loci. Nat Rev Microbiol.

[27] Van Melderen L, Saavedra De Bast M (2009). Bacterial toxin-antitoxin systems: more than selfish entities?. PLoS Genet.

[28] Makarova KS, Wolf YI, Koonin EV (2009). Comprehensive comparative-genomic analysis of type 2 toxin-antitoxin systems and related mobile stress response systems in prokaryotes. Biol Direct.

[29] Makarova KS, Wolf YI, Snir S, Koonin EV (2011). Defense islands in bacterial and archaeal genomes and prediction of novel defense systems. J Bacteriol.

[30] Makarova KS, Wolf YI, Koonin EV (2013). The basic building blocks and evolution of CRISPR-cas systems. Biochem Soc Trans.

[31] Koonin EV, Makarova KS (2013). CRISPR-Cas: evolution of an RNA-based adaptive immunity system in prokaryotes. RNA Biol.

[32] Frank SA (1998). Inducible defence and the social evolution of herd immunity. Proc Biol Sci.

[33] Frank SA (2000). Specific and non-specific defense against parasitic attack. J Theor Biol.

[34] Andre JB, Ferdy JB, Godelle B (2003). Within-host parasite dynamics, emerging trade-off, and evolution of virulence with immune system. Evolution.

[35] Alizon S, van Baalen M (2008). Multiple infections, immune dynamics, and the evolution of virulence. Am Nat.

[36] Weinberger AD, Wolf YI, Lobkovsky AE, Gilmore MS, Koonin EV (2012). Viral diversity threshold for adaptive immunity in prokaryotes. MBio.

[37] Iranzo J, Lobkovsky AE, Wolf YI, Koonin EV (2013). Evolutionary dynamics of the prokaryotic adaptive immunity system CRISPR-Cas in an explicit ecological context. J Bacteriol.

[38] Lennon JT, Khatana SA, Marston MF, Martiny JB (2007). Is there a cost of virus resistance in marine cyanobacteria?. ISME J.

[39] Quance MA, Travisano M (2009). Effects of temperature on the fitness cost of resistance to bacteriophage T4 in Escherichia coli. Evolution.

[40] Palmer KL, Gilmore MS (2010). Multidrug-resistant enterococci lack CRISPR-cas. MBio.

[41] Koskella B, Lin DM, Buckling A, Thompson JN (2012). The costs of evolving resistance in heterogeneous parasite environments. Proc Biol Sci.

[42] Weinberger AD, Gilmore MS (2012). CRISPR-Cas: to take up DNA or not-that is the question. Cell Host Microbe.

[43] Jiang W, Maniv I, Arain F, Wang Y, Levin BR, Marraffini LA (2013). Dealing with the evolutionary downside of CRISPR immunity: bacteria and beneficial plasmids. PLoS Genet.

[44] Heilmann S, Sneppen K, Krishna S (2012). Coexistence of phage and bacteria on the boundary of self-organized refuges. Proc Natl Acad Sci U S A.

[45] Debarre F, Lion S, van Baalen M, Gandon S (2012). Evolution of host life-history traits in a spatially structured host-parasite system. Am Nat.

[46] Fukuyo M, Sasaki A, Kobayashi I (2012). Success of a suicidal defense strategy against infection in a structured habitat. Sci Rep.

[47] Restif O, Koella JC (2004). Concurrent evolution of resistance and tolerance to pathogens. Am Nat.

[48] Nunez JK, Kranzusch PJ, Noeske J, Wright AV, Davies CW, Doudna JA (2014). Cas1-Cas2 complex formation mediates spacer acquisition during CRISPR-Cas adaptive immunity. Nat Struct Mol Biol.

[49] He F, Chen L, Peng X (2014). First Experimental Evidence for the Presence of a CRISPR Toxin in Sulfolobus. J Mol Biol.

[50] Greischar MA, Koskella B (2007). A synthesis of experimental work on parasite local adaptation. Ecol Lett.

[51] Vos M, Birkett PJ, Birch E, Griffiths RI, Buckling A (2009). Local adaptation of bacteriophages to their bacterial hosts in soil. Science.

[52] Hargreaves KR, Flores CO, Lawley TD, Clokie MR (2014). Abundant and diverse clustered regularly interspaced short palindromic repeat spacers in Clostridium difficile strains and prophages target multiple phage types within this pathogen. MBio.

[53] Samson JE, Magadan AH, Sabri M, Moineau S (2013). Revenge of the phages: defeating bacterial defences. Nat Rev Microbiol.

[54] Hamilton WD (1964). The genetical evolution of social behaviour. I. J Theor Biol.

[55] Griffin AS, West SA, Buckling A (2004). Cooperation and competition in pathogenic bacteria. Nature.

[56] Nowak MA, Tarnita CE, Antal T (2010). Evolutionary dynamics in structured populations. Philos Trans R Soc Lond B Biol Sci.

[57] Chao L, Levin BR (1981). Structured habitats and the evolution of anticompetitor toxins in bacteria. Proc Natl Acad Sci U S A.

[58] Lion S, Baalen M (2008). Self-structuring in spatial evolutionary ecology. Ecol Lett.

[59] Kummerli R, Griffin AS, West SA, Buckling A, Harrison F (2009). Viscous medium promotes cooperation in the pathogenic bacterium Pseudomonas aeruginosa. Proc Biol Sci.

[60] Refardt D, Bergmiller T, Kummerli R (2013). Altruism can evolve when relatedness is low: evidence from bacteria committing suicide upon phage infection. Proc Biol Sci.

[61] Dimitriu T, Lotton C, Benard-Capelle J, Misevic D, Brown SP, Lindner AB (2014). Genetic information transfer promotes cooperation in bacteria. Proc Natl Acad Sci U S A.

[62] Lion S, Boots M (2010). Are parasites ''prudent'' in space?. Ecol Lett.

[63] Hay S, Kannourakis G (2002). A time to kill: viral manipulation of the cell death program. J Gen Virol.

[64] Postigo A, Ferrer PE (2009). Viral inhibitors reveal overlapping themes in regulation of cell death and innate immunity. Microbes Infect.

[65] Agol VI, Gmyl AP (2010). Viral security proteins: counteracting host defences. Nat Rev Microbiol.

[66] Kaufmann G (2000). Anticodon nucleases. Trends Biochem Sci.

[67] van Baalen M (1998). Coevolution of recovery ability and virulence. Proc Biol Sci.

[68] Roy BA, Kirchner JW (2000). Evolutionary dynamics of pathogen resistance and tolerance. Evolution.

[69] Dupuis ME, Villion M, Magadan AH, Moineau S (2013). CRISPR-Cas and restriction-modification systems are compatible and increase phage resistance. Nat Commun.

[70] Atkinson S, Williams P (2009). Quorum sensing and social networking in the microbial world. J R Soc Interface.

[71] Hartmann A, Schikora A (2012). Quorum sensing of bacteria and trans-kingdom interactions of N-acyl homoserine lactones with eukaryotes. J Chem Ecol.

[72] Kovacs AT (2014). Impact of spatial distribution on the development of mutualism in microbes. Front Microbiol.

